# Comparison of Complications of Non‐Resorbable (Permanent) Fillers and Resorbable Fillers

**DOI:** 10.1111/jocd.16553

**Published:** 2024-09-23

**Authors:** Tom S. Decates, Valerie Vroege, Merel A. Hamer, Peter Velthuis

**Affiliations:** ^1^ Department of Dermatology Erasmus Medical Center Rotterdam The Netherlands

**Keywords:** complication, cosmetic dermatology, fillers, hyaluronic acid fillers, permanent fillers

To the Editor

Filler treatments are a popular cosmetic procedure used worldwide. In 2020, the number of soft tissue filler (STF) treatments performed by plastic surgeons in the United States was estimated at 1.3 million, with a revenue of almost $ 1 billion [1]. Although there are many different classifications of soft tissue fillers, for the purpose of adverse event description they are mostly classified by their biodegradability into non‐resorbable (permanent) and resorbable fillers [2]. Permanent fillers remain in situ indefinitely. These have been used in the past for many years but have been banned in many countries in the past years (in the Netherlands since 2015) [3]. Permanent fillers have been tested and initially found suitable for medical use. But many non‐descript, non‐medical substances are injected around the world. Frequently used examples are medical‐grade silicone, polyalkylimide, polyacrylamide, and methacrylate fillers [4]. Until 2015, different types of permanent fillers were injected, ending up in an adverse event rate of up to 50% [5]. Aside from the direct harm to the patient, leading to lifelong physical and mental scaring, complications constitute a considerable financial burden to the healthcare system. In 1999, it was estimated that the total costs of preventable adverse events in the United States lie between $17 and $29 billion annually [6].

Since 2011, the Dermatology department of Erasmus Medical Centre in Rotterdam (the Netherlands) operates an out‐patient clinic for soft tissue filler complications. Patients are referred by general practitioners, dermatologists, or other medical specialists. Complications range from infections and noduli to pain, hair loss, or simply cosmetic complaints such as a small asymmetry after lip filler treatment. Ultrasound imaging is often used as a tool to determine the type of filler injected as well as its location in the skin. After diagnosing the problem, patients are treated with a suitable treatment for their complication and filler type.

Between 2011 and 2016, 401 new patients have presented at the Erasmus MC Dermatology Department with filler complications [7]. Patients were categorized by type of filler, showing 77.6% of these patients were concerned with permanent fillers. The current study aims to repeat the Schelke et al. study [7] for the year 2022 in order to portray any shift in the types of fillers responsible for complications in recent years.

In this retrospective observational study, the Electronic Health Reports (EHR) of all new patients at the out‐patient clinic for filler complications in 2022 were examined. For each patient, the type of filler causing the complication was extracted as being either non‐resorbable (permanent) or resorbable. In total, 316 patients were included in this study. Of the 316 new patients seen at the clinic, the largest group of patients had used a non‐resorbable filler (*N* = 160, Table 1). The hyaluronic acid (HA) filler group was the second largest group, with a total of 131 patients.

**TABLE 1:** Percentages of non‐resorbable versus resorbable fillers at the out‐patient clinic in 2011–2016 and 2022.

Type of filler	Number of patients (%)	
	2011–2016	2022
Non‐resorbable	311 (77.6)	160 (50.6)
Resorbable	75 (18.7)	139 (44)
Hyaluronic acid	55 (13.7)	131 (41.5)
Bio‐Stimualory	14 (3.5)	8 (2.5)
CaHa	13 (3.2)	7 (2.2)
PLLA	1 (0.3)	1 (0.3)
Collagen	6 (1.5)	0
Unkown	0	5 (1.6)
Other	15 (3.7)	12 (3.8)

The results in Figure 1 show the outcomes of the comparison between the numbers in this study and those in the study by Schelke et al. When comparing the percentages of complications caused by each type of filler, we found that the relative number of complications caused by non‐resorbable fillers has decreased in recent years, whereas those caused by resorbable HA fillers have increased.

**FIGURE 1:**
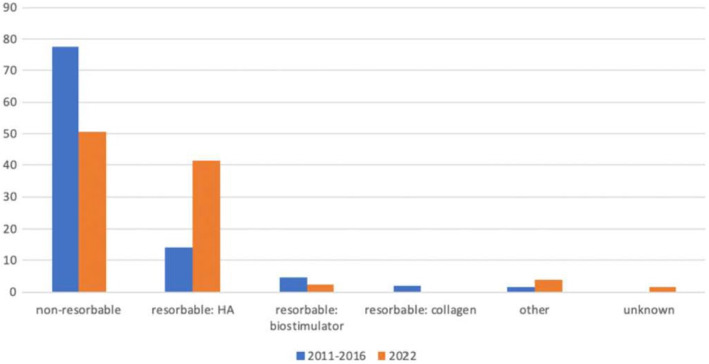
Percentage of filler types causing complications 2011–2016 compared to 2022.

In both cohorts, most individuals presented with complications caused by non‐resorbable (permanent) fillers, which were clinically expressed as late‐onset inflammation. In 2011–2016, complications caused by non‐resorbable fillers accounted for 77.6% of the total patient population. In 2022, this proportion has dropped to 50.6%, a difference of 27% (95% CI 20.1–33.9).

As mentioned above, non‐resorbable fillers often cause late‐onset inflammation. A report by IQUAM in July of 2006 stressed the association between non‐resorbable implants and risks of infection, granuloma formation, and long‐term irreversible complications [8]. Examples as the capacity to migrate, as well as the early or delayed foreign body reaction associated with silicone injections. This is in line with the high incidence of late‐onset inflammation caused by non‐resorbable fillers observed in this study.

The use of non‐resorbable fillers has been officially forbidden in the Netherlands since January 2015 [3]. However, new cases are still referred to us because of the long delay in the development of filler complications in this group and also because of the influx of new cases treated with permanent fillers outside the Netherlands. In particular, many cases of buttock fillers from the Caribbean and facial fillers from the Middle East present themselves in the clinic.

Non‐resorbable (permanent) fillers still account for most of the complications referred to our outpatient clinic. These fillers can cause long‐term irreversible complications (scars, dents, and edema) leading to lifelong physical and psychological sequels, as well as a considerable financial burden on the healthcare system. More cohort studies are required to assess the complication profiles of non‐resorbable fillers, both quantitatively and qualitatively, in order to raise awareness in both patients and doctors.

## Ethics Statement

Research concerning anonymously non‐traceable data does not require approval by an ethics committee according to Dutch law (WMO).

## Conflicts of Interest

The authors declare no conflicts of interest.

## Data Availability

The data that support the findings of this study are available on request from the corresponding author. The data are not publicly available due to privacy or ethical restrictions.
